# Supplementary material for: Association of SNP rs5069 in APOA1 with Benign Breast Diseases in a Mexican Population
Source: Genes (Basel). 2022 Apr 22;13(5):738. doi: 10.3390/genes13050738 (PMC9141650; doi:10.3390/genes13050738)
Supplement: Supplementary file 1 [file genes-13-00738-s001.zip › genes-1672176-supplementary.pdf]

Table S1. Diagnosis and age distribution for breast cancer and benign breast disease groups.

**Breast Cancer group**			
**Diagnosis**	**n**	**Age**	**n**
Invasive ductal carcinoma	22	30 – 39	4
Ductal carcinoma	6	40 – 49	14
Invasive lobular carcinoma	4	50 – 59	6
Lobular carcinoma	2	60 – 69	8
Invasive carcinoma	2	> 70	3
Mucinous carcinoma	1	N/A	4
Residual carcinoma	1		
Carcinoma	1		
**Benign Breast Disease group**			
**Diagnosis**	**n**	**Age**	**n**
Fibrocystic breast disease	8	<30	2
Intracanicular and pericanicular fibroadenoma	2	30 – 39	7
Angiolipoma	1	40 – 49	4
Hyperplasia	1	50 – 59	3
Stromal fibrosis	1	60 – 69	1
**Negative diagnosis for malignancy**	**6**	**N/A**	**2**

Table S2. PCR-RFLP conditions for the analyzed polymorphisms

Gene	Primer	Sequence (5' – 3')	Amplicon size (pb)	TA(°C)	Enzyme	Restriction fragments (bp) [a]
**APOA1** rs670 & rs5069	MSP1	GGACCAGTGAGCAGCAACA[b]	220[c]	60°C	MspI	23, 114, 46, 37[d]
	MSP2	ACAGAGCGGGAGAAGACCT				
rs670	MSP1	GGACCAGTGAGCAGCAACA[b]	118[e]	58°C		23, 95
	MSP3	CCTCCTTCTCGCAGTCTCTAAG				
rs5069	MSP4	GCTTAGAGACTGCGAGAAGGA	125[c, f]	58°C		42,46, 37
	MSP2	ACAGAGCGGGAGAAGACCT				
**APOB**	rs693 f	AAGCCTACAGGACACCAA	167	52°C	XbaI	100, 67
	rs693 r	GAGACAGGTATCGTTGAAGT				
	rs104203-f	ACCTACCAAGAGTGGAAGGACAA	167	56°C	EcoRI	124, 43
	rs1042031-r	TCAATGAGTGAGTCAATCAGATGCT				

TA=annealing temperature

[a]Fragments are listed in order of site cuts

[b]Primer designed by Smith et al., 1992

[c]Includes a non-polymorphic restriction site for *Msp*I.

[d]Fragments obtained in presence of the MspI restriction site at −75 bp (G allele) for rs670 and at +83 bp (C allele) for rs5069 in the 220 bp amplicon.

[e]Amplicon obtained with MSP1 (fw) and MSP3 (rv) for rs670.

[f]Amplicon obtained with MSP4 (fw) and MSP2 (rv) for rs5069.

Table S3. Genotype independent testing against reference genotype for rs670, rs5069, rs693 in all groups

SNP (Gene)	Genotype	BCa vs GP	BBD vs GP	BCa vs BBD
		p	*p*	*p*
rs670	GA vs GG	0.2483	0.5758	1
(APOA1)	AA vs GG	0.5264	0.027*	0.2481
rs5069	CT vs CC	0.8344	0.0116*	0.0713
(APOA1)	TT vs CC	0.5833	0.2669	1
rs693	CT vs CC	0.3368	0.594	1
(APOB)	TT vs CC	0.4992	0.6832	0.3254
rs1042031	GA vs GG	0.1264	0.3823	0.1089
(APOB)	AA vs GG	1	0.5972	1

Fisher's exact test was used to determine significant differences between groups. BCa: Breast cancer group; BBD: Benign Breast disease group; GP: General population group. *p<0.05
